# A Genotype-First Approach for the Molecular and Clinical Characterization of Uncommon De Novo Microdeletion of 20q13.33

**DOI:** 10.1371/journal.pone.0012462

**Published:** 2010-08-27

**Authors:** Ryan N. Traylor, Damien L. Bruno, Trent Burgess, Robert Wildin, Anne Spencer, Devika Ganesamoorthy, David J. Amor, Matthew Hunter, Michael Caplan, Jill A. Rosenfeld, Aaron Theisen, Beth S. Torchia, Lisa G. Shaffer, Blake C. Ballif, Howard R. Slater

**Affiliations:** 1 Signature Genomic Laboratories, Spokane, Washington, United States of America; 2 Victorian Clinical Genetics Services, Murdoch Children's Research Institute, Royal Children's Hospital, Parkville, Victoria, Australia; 3 Department of Pediatrics, University of Melbourne, Royal Children's Hospital, Parkville, Victoria, Australia; 4 St. Luke's Regional Medical Center, Boise, Idaho, United States of America; 5 Northshore University Healthsystem, Pritzker School of Medicine, University of Chicago, Chicago, Illinois, United States of America; Ohio State University Medical Center, United States of America

## Abstract

**Background:**

Subtelomeric deletions of the long arm of chromosome 20 are rare, with only 11 described in the literature. Clinical features of individuals with these microdeletions include severe limb malformations, skeletal abnormalities, growth retardation, developmental and speech delay, mental retardation, seizures and mild, non-specific dysmorphic features.

**Methodology/Principal Findings:**

We characterized microdeletions at 20q13.33 in six individuals referred for genetic evaluation of developmental delay, mental retardation, and/or congenital anomalies. A comparison to previously reported cases of 20q13.33 microdeletion shows phenotypic overlap, with clinical features that include mental retardation, developmental delay, speech and language deficits, seizures, and behavior problems such as autistic spectrum disorder. There does not appear to be a clinically recognizable constellation of dysmorphic features among individuals with subtelomeric 20q microdeletions.

**Conclusions/Significance:**

Based on genotype-phenotype correlation among individuals in this and previous studies, we discuss several possible candidate genes for specific clinical features, including *ARFGAP1*, *CHRNA4* and *KCNQ2* and neurodevelopmental deficits. Deletion of this region may play an important role in cognitive development.

## Introduction

Because molecular cytogenetic techniques such as microarray-based comparative genomic hybridization (aCGH) can identify chromosome abnormalities in individuals with nonspecific symptoms, such as developmental delay, without the need for clinical suspicion of a specific disorder, these techniques have enabled a “genotype-first” approach to the genotype-phenotype characterization of rare chromosome abnormalities. In such an approach, individuals with similar genotypes, or genetic constitutions, can be identified and examined to determine a common phenotype, or collection of clinical features [1]. High-resolution microarray analysis also can detect small DNA imbalances that may reveal the causative genes for specific clinical features, which can can aid diagnosis and prognosis.

Subtelomeric deletions of the long arm of chromosome 20 are rare, with only 11 described in the literature [2]–[11]. Of these previously reported subtelomeric deletions, five individuals have been well characterized by microarray analysis [7]–[9], [11]. Clinical features of individuals with these microdeletions include severe limb malformations, skeletal abnormalities, growth retardation, developmental and speech delay, mental retardation, and seizures. There does not appear to be a clinically recognizable constellation of dysmorphic features among individuals with subtelomeric 20q microdeletions.

In this study we report six additional individuals with microdeletions at 20q13.33 and compare their phenotypes with previously reported individuals with microdeletions at 20q13.33 microdeletion. Among these individuals are several common clinical features including mental retardation, developmental delay, speech and language deficits, seizures, and behavioral problems.

## Results

### Molecular analysis

**Figure 1:**
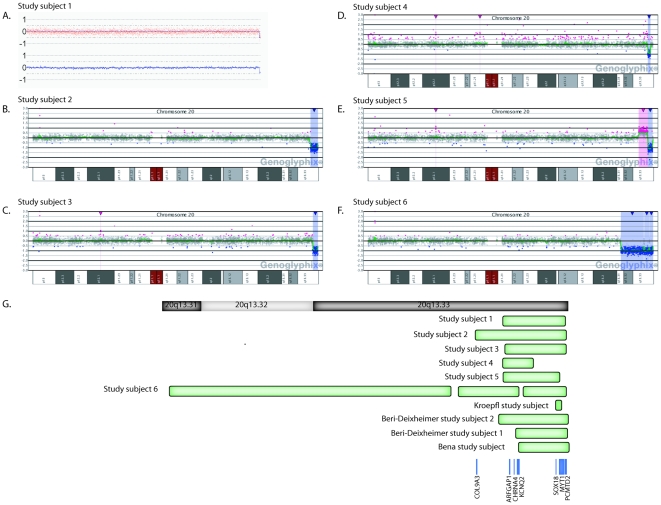
Microarray-based characterization of microdeletions at 20q13.33. (**A–F**) Microarray results for study subjects 1–6, respectively. Study subject 1 was analyzed using a SNP microarray; study subjects 2–6 were analyzed using an oligonucleotide CGH-based array. For study subject 1, Copy Number Analyser for GeneChip (CNAG) version 3.0 [30] was used for the analysis. For study subjects 2–6, results were visualized using custom aCGH analysis software (Genoglyphix; Signature Genomic Laboratories). Study subject 5 had a concurrent duplication at 20q13.33 (**E**). Probes are arranged with the most proximal 20q13.31 probes on the left and the most distal 20q13.33 probes on the right. (**G**) Schematic representation of deletions in study subjects 1–6 and in previously reported study subjects. Blue boxes represent genes of interest within the region.

We identified six individuals with microdeletions at 20q13.33 ranging in size from ∼561 kb to ∼6.8 Mb (Table 1). Terminal deletions were found in study subjects 1–3 and 6 and a interstitial deletions was identified in study subject 4 (Figure 1). Study subject 5 had a complex alteration with a duplication and deletion, and study subject 6 had a complex alteration with three noncontiguous interstitial deletions. The deletions in study subjects 3–6 were visualized by fluorescence *in situ* hybridization (FISH). Parental FISH testing for study subjects 1, 2, and 5 was normal; thus, these deletions are apparently *de novo* in origin. The 20q13.33 deletion in study subject 4 was also identified in the father.

**Table 1:** Molecular findings of study subjects with 20q13.33 microdeletion.

	Study subject 1	Study subject 2	Study subject 3	Study subject 4	Study subject 5	Study subject 6
Sex	Male	Female	Male	Male	Male	Female
GENETIC FINDINGS						
Inheritance	*De novo*	*De novo*	Unknown	Paternal	*De novo*	Unknown
Deletion Size	∼1.1 Mb	∼1.61 Mb	∼1.08 Mb	∼560 kb	∼1.0 Mb	∼6.8 Mb
ISCN (UCSC hg18)	arr 20q13.33(61,246,745–62,376,958)x1 dn	arr 20q13.33(60,760,865–62,379,119)x1 dn	arr 20q13.33(61,290,900–62,379,119)x1	arr 20q13.33(61,238,755–61,800,435)x1	arr 20q13.33(59,234,582–61,238,814)x3,20q13.33(61,264,602–62,266,519)x1	arr 20q13.33(55,328,239–60,337,959)x1,20q13.33(60,462,519–61,543,335)x1,20q13.33(61,617,738–62,379,119)x1
GEO accession number	GSM541664	GSM537891	GSM537892	GSM537893	GSM537894	GSM537895
OMIM Gene Content	24 deleted, including ARFGAP1, CHRNA4, KCNQ2,SOX18, MYT1	36 deleted, including COL9A3, ARFGAP1, CHRNA4, KCNQ2, SOX18, MYT1	24 deleted, including ARFGAP1, CHRNA4, KCNQ2, SOX18, MYT1	12 deleted, including ARFGAP1, CHRNA4, KCNQ2	25 genes within duplication; 23 genes within deletion including ARFGAP1, CHRNA4, KCNQ2, SOX18	80 deleted, including COL9A3, ARFGAP1, CHRNA4, KCNQ2, SOX18, MYT1

### Clinical summaries

**Figure 2:**
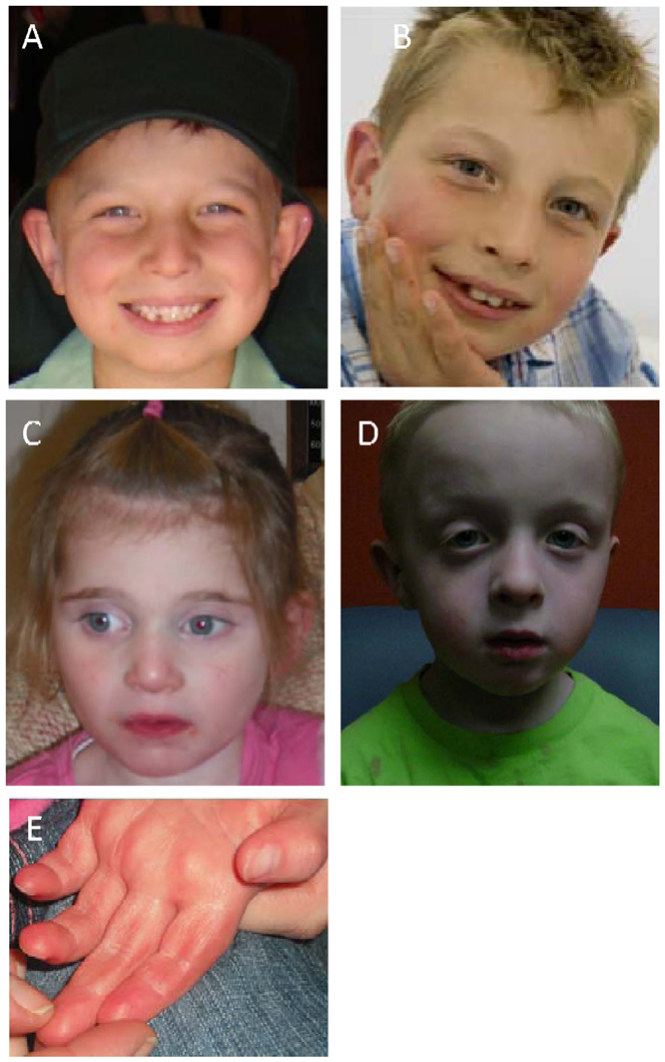
Dysmorphic features in individuals with microdeletions at 20q13.33. (**A,B**) Study subject 1 at age 8 years. No dysmorphic facial features were noted. (**C**) Study subject 2 at age 4 years. Note bitemporal narrowing, bulbous nose, and upslanting palpebral fissures. (**D**) Study subject 3 at age 3 years 3 months. Note bifrontal prominence, prominent forehead, triangular shape, mild hypertelorism, epicanthal folds, and ptosis. (**E**) Hand of study subject 2.

Study subject 1 is a 9-year-old male, the first of two children born to nonconsanguineous parents. He was born at 37 weeks gestation by Cesarean section following a normal pregnancy. His birth weight was in the 10–25^th^ percentile. Neonatally he was slow to establish breast feeding. He was noted to have a single generalized seizure that was not associated with fever at 6 months of age. An EEG was normal and antiepileptic treatment was not required. Developmental milestones were mildly delayed: he sat at 9 months, crawled at 10 months and walked at 15½ months. At 18 months he was diagnosed with low muscle tone, motor dyspraxia and global developmental delay. An MRI performed at 7 years of age showed prominent perivascular spaces bilaterally with some cystic dilatation in the left parietal lobe. A WISC assessment at age 9 years showed a global IQ of 40. His hearing and vision are normal, as are his height, weight and head circumference (HC). He did not have any dysmorphic features (Figure 2A–B).

Study subject 2 is a 4-year-old female born at term following a normal pregnancy. Her birth weight was in the 10^th^ percentile. She is the second of three children born to healthy nonconsanguineous parents, with no family history of note, apart from a single febrile convulsion in the mother. Apgars were 6 at 1 minute and 7 at 5 minutes. Seizures began at 2 weeks of life and have since evolved into complex partial seizures, which have been ongoing and difficult to control with medication. Development has been globally delayed, particularly in speech and language. At 4 years of age she has minimal expressive language, repetitive behaviors, and minimal social interactions. She is not toilet trained and cannot feed herself. Physical examination revealed minor dysmorphism consisting of relatively long and mildly upslanting palpebral fissures, mild bitemporal narrowing, and a slightly bulbous nose (Figure 2C). Her height is at the 1st percentile, and weight and HC are normal. Metabolic investigations were normal. An MRI scan showed a structurally normal brain with delayed myelination in the temporal lobes. Audiology and ophthalmology assessments were normal.

Study subject 3 is a 3-year, 3-month-old male, the third of three sons born to healthy nonconsanguineous parents; his two brothers are healthy. He was born at 35 weeks gestation by emergency Cesarean section because of a breech presentation and severe oligohydramnios. Birth weight was <5^th^ percentile. He had failure to thrive in infancy. He has developmental delay, clubfoot, and congenital dislocation of the right hip with chief complaint of small lower legs and multiple congenital anomalies. At 6 months he was treated for the hip dislocation with spica cast and surgery. Muscle mass and tone is slightly decreased, particularly in the lower legs with a deficit of strength. Gait shows some ballistic movements of the lower legs. Dysmorphic features include pale complexion, bifrontal prominence, prominent forehead, and prominent parietal areas producing a triangular head and face. He had epicanthal folds, down-slanting palpebral fissures, symmetric facial hypotonia, slight hypertelorism, ptosis, low-set posteriorly rotated ears with a thickened helix and prominent antihelix and crus, narrow-tipped nose with decreased nasolabial folds and small alae nasi (Figure 2D). He has significant developmental delay at the age of 32 months, with his developmental level at 12-15 months. Both his receptive and expressive speech are delayed, and he has only a few words. He has motor delays, evident by a discoordinated gait. He does not have seizures. He has a hypoplastic left nipple although the areola is of normal size with the nipple eccentric. He is at male stage Tanner 1 with hint of hypospadias with a hypoplastic, flat scrotum. Both testes are palpated, the left low in the canal and the right in the canal but moving into the scrotum easily. His back is straight with a mildly asymmetric sacral dimple. MRI of lumbar spine and partial MRI of entire spine were normal. His fingers are slightly long and tapering. Lower legs have mild shortening and thinning without apparent bony anomalies. Feet are without arches and with a prominent heel, with the large toes pronated and laterally angulated.

Limited information is available for study subjects 4 and 5. Study subject 4 is a 9-year-old male referred for microarray-based comparative genomic hybridization (aCGH) for developmental delay, dysmorphic features, and seizure disorder. Study subject 5 is a 23-month-old male referred for aCGH for developmental delay and seizure disorder.

Study subject 6 was an 8-month-old female delivered at 30 weeks gestation by Caesarean section for fetal distress and oligohydramnios. Birthweight was 1335 g (33^rd^ percentile), length was 37 cm (16^th^ percentile), and head circumference was 27.5 cm (26^th^ percentile). There were no obvious dysmorphic features other than low-set, posteriorly rotated ears. She required respiratory support for two days for mild surfactant deficiency, and then was extubated and advanced on enteral feedings. Due to the oligohydramnios without evidence of prolonged rupture of membranes, chromosomal analysis was done that identified a deletion in the terminal end of chromosome 20, and CGH was sent for further evaluation. Her hospital course was complicated by recurrent and severe thrombocytopenia of unknown etiology requiring multiple platelet transfusions and intravenous IV IgG, mild hydronephrosis, an echocardiogram that showed a hypoplastic aortic arch, perimembranous VSD, and secundum ASD, and a head ultrasound that demonstrated asymmetry of ventricular size without associated abnormalities. On hospital day number 22, she developed acute abdominal distention with hematochezia, and abdominal radiographs revealed extensive pneumatosis intestinalis with dilated loops of bowel. She deteriorated over six hours despite full resuscitative efforts, and demonstrated severe metabolic acidosis, hypotension, capillary leak, poor perfusion, and respiratory failure. Surgical exploration confirmed the diagnosis of necrotizing enterocolitis with total necrosis of the entire bowel, and she expired shortly thereafter.

## Discussion

We have identified six individuals with microdeletions at the subtelomeric region of 20q13.33 among individuals referred for molecular cytogenetic testing. A comparison of the clinical features of the four individuals in our study for whom detailed information was available with those of previously published individuals with microdeletions of 20q13.33 identified by aCGH shows several recurring features, including cognitive and language deficits (Table 2). Of note, study subject 6, who had a complex alteration with three noncontiguous interstial deletions extending 6.8 Mb, presented with the most severe phenotype but no apparent dysmorphic features. The severity of the phenotype may be owing to the complexity of the rearrangement, the breakpoint locations, and gene content. No additional reports of a similar phenotype or early death have been described among individuals with loss of 20q13.33 [7]–[9].

**Table 2:** Clinical features of study subjects with 20q13.33 microdeletions.

	Study subject 1	Study subject 2	Study subject 3	Study subject 6	Beri-Deixheimer study subject 1[7]	Beri-Deixheimer study subject 2[7]	Bena study subject [9]	Kroepfl study subject [8]
Sex	Male	Female	Male	Female	Female	Female	Female	Female
Age	9y	4y	3y 3mo	8mo*	7y	4y	4y	30mo
Dysmorphic Features	-	Bitemporal narrowing, upslanting palpebral fissures, bulbous nose	Bifrontal prominence, prominent forehead, triangular shape, downslanting palpebral fissures, mild hypertelorism, epicanthal folds, ptosis, narrow-tipped nose, decreased nasolabial folds, small alae nasi	Low-set, posteriorly rotated ears	Microcephaly, brachycephaly, prominent metopic suture, temporal narrowing, upslanting palpebral fissures, mild hypertelorism, nystagmus, large anteverted nostrils, thin upper lip, downturned corners of the mouth	Trigonocephaly, temporal narrowing, epicanthic folds, mild hypertelorism	Convergent strabismus, hyperopia, astigmatism of right eye	Upslanting palpebral fissures, slightly arched eyebrows, bulbous nose, thin upper lip, slight cupids bow, long philtrum
Psychomotor and behavioral development	Global IQ 40, motor dyspraxia, hypotonia	Global developmental delay, minimal expressive language, repetitive behavior, minimal social interaction	Severe developmental delay, expressive and receptive delay, destructive and hyperactive behavior, mild hypotonia, discoordinated gait		Mild global delay, walked 19 mo, speech delay	Severe global delay, sat 21 mo, poor social interaction, autism, sleep disturbances	Learning difficulties, speech delay	Severe global delay, speech delay, mild hypotonia
Neurology	Single seizure without fever at 6 mo	Seizure onset at 2 weeks, evolved into complex partial seizures	-	-	-	Single episode of convulsion without fever at age 2 mo	Normal	Normal
Brain MRI	N/A	Structurally normal with delayed myelination	N/A		Normal	Thin corpus callosum at age 10 mo	N/A	Normal
Other			Congenital dislocation of hip and clubfoot				Joint laxity with flat feet	

“+”: feature present; “-”: feature absent; DD: developmental delay; DF: dymorphic features; MRI: magnetic resonance imaging; NA: not available; *patient is deceased.

Among individuals with 20q13.33 microdeletion, several have seizures (study subjects 1 and 2 in this study and Beri-Deixheimer study subject 2 [7]) and behavior problems (study subjects 2 and 3 in this study and Beri-Deixheimer study subject 2 [7]). Behavioral problems identified in the current cohort include autistic behavior and minimal social interactions (study subject 2) and hyperactive, destructive behaviors (study subject 3). Behaviors noted in the literature include poor social interaction (Beri-Deixheimer study subject 2 [7] and Kroepfl study subject 1 [8]) and autism (Beri-Deixheimer study subject 2 [7]). Additional behaviors reported in individuals with microdeletions at 20q13.33 include anxiety (Bena study subject [9]) and sleep disturbances (Beri-Deixheimer study subject 2 [7]). As previously suggested, there does not appear to be a clinically recognizable constellation of dysmorphic features among individuals with subtelomeric 20q microdeletions.

Seizures have been reported for several individuals with microdeletions encompassing 20q13.3 [2], [7], [12]. Among them, two individuals with large terminal deletions of 20q presenting with severe generalized tonic seizures [2], [12]. Study subject 2 reported by Beri-Deixheimer and colleagues [7] suffered from a single episode of convulsion without fever at 2 months of age. Of the current cohort, study subject 1 had a single seizure event at the age of 6 months, and study subject 2 had seizure onset at 2 weeks of life that progressed into complex partial seizures. Study subjects 4 and 5 were referred for genetic evaluation of seizures, although further clinical information is not available for either individual. Several genes on 20q13.33 are associated with epilepsy. Mutations in *KCNQ2* and *CHRNA4* have been identified in individuals with benign familial neonatal convulsions (BFNC) and autosomal dominant nocturnal frontal lobe epilepsy (ADNFLE), respectively [13]–[15]. All of the study subjects molecularly characterized in this current report include loss of the gene group *ARFGAP1, CHRNA4,* and *KCNQ2*. Mouse models of *Stz1* mutants, harboring a 300 kb deletion on mouse chromosome 2 including *Kcnq2, Arfgap1*, and *Chrna4*, demonstrate an increased susceptibility to seizures [16]. *ARFGAP1* influences cell growth, migration, and membrane traffic by sorting cargo and driving vesicle formation at the Golgi apparatus [17]. *CHRNA4* encodes alpha-4 nicotinic acetylcholine receptor subunits and knockout mice show heightened anxiety-like behavior [16]. Potassium ion channels are involved in mammalian epilepsy, and *Kcnq2* heterozygous mutant mice demonstrate its role in the control of neuronal excitability [14]. Study subjects 3 and 6 do not present with seizures which suggests deletion of these genes may show incomplete penetrance. Additional study subjects with non-recurrent deletions encompassing *ARFGAP1, CHRNA4*, and *KCNQ2* may provide further detailed genotype-phenotype correlations with respect to this gene group's role in brain development and function.

The smallest 20q13.33 microdeletion, report by Kroepfl and colleagues [8], is a subtelomere microdeletion encompassing only two genes, myelin transcription factor 1 (*MYTI*) and protein-L-isoaspartate (D-aspartate) O-methyltransferase domain containing 2 (*PCMTD2*) (Figure 1). *MYT1* regulates neuronal transcription and is involved in the proliferation and differentiation of oligodendrocytes, cells that form the myelin sheath in the central nervous system [18]. *PCMTD2* has not yet been well described and has no animal model. *MYT1* is deleted in all subjects in the current study except study subject 4 and in all recently reported individuals with microdeletions at 20q13.33 [7]–[9]. Of those described, impaired psychomotor and behavioral development have been observed [7]–[9]; paired with data on the gene's function, *MYT1* is a good candidate gene for further study to elucidate its involvement in brain function and any associated neurological disorders influencing cognitive development.

Among the other genes with known functions in the deletion region is *SRY-box 18* (*SOX18*), homozygous missense mutations and heterozygous nonsense mutations of which have been associated with recessive and dominant forms of hypotrichosis-lymphedema-telangiectasia syndrome [19]. *SOX18* is known to play a role in blood vessel development, hair follicle formation, and lymphangiogenesis in mice [20]–[22]. The lack of features associated with hypotrichosis-lymphedema-telangiectasia syndrome in this and previous reports suggests that deletions of *SOX18* may cause a milder phenotype than mutations (Table 2). Other recognized genes within the 20q13.33 region include *collagen Type IX, alpha-3* (*COL9A3*), splice-site mutations of which have been reported in families with multiple epiphyseal dysplasia [23]–[25]. Clinical features observed within these families include stiffness and pain with limited extension of joints in childhood, with some requiring surgery in adulthood [23]–[25]. Subjects 2 and 6 in this study have deletions that encompass *COL9A3*, but the lack of clinical features reminiscent of multiple epiphyseal dysplasia suggests deletion of *COL9A3* does not cause the syndrome.

Microdeletions at 20q13.33 are associated with a constellation of clinical features that includes mental retardation, developmental delay, speech and language deficits, seizure, and behavioral problems such as autistic spectrum disorder. However, there is no pattern of abnormalities, craniofacial, behavioral, or otherwise, that would arouse clinical suspicion of a 20q13.33 microdeletion; thus, molecular cytogenetic techniques such as aCGH that do not rely on clinical suspicion of a specific disorder are useful for diagnosis of individuals with these imbalances. The identification of further individuals with overlapping 20q13.33 deletions will help elucidate the roles that individual genes within this region have on the abnormal phenotypes observed in these individuals.

## Materials and Methods

### Study subject ascertainment

Study subjects 1 and 2 were ascertained by Genetic Health Services Victoria in Melbourne, Australia. Study subjects 3–6 were ascertained by Signature Genomic Laboratories. For study subjects 1 and 2, written informed consent using a Genetic Health Services Victoria Institutional Review Board (IRB)-approved consent form was obtained to perform high-resolution microarray-based molecular cytogenetic testing. For study subjects 3–6, written informed consent using an IRB-Spokane-approved consent form was obtained to perform high-resolution microarray-based molecular cytogenetic testing. This study was approved by the respective IRBs. IRB-approved written consent was also obtained from study subjects 1 and 3 to publish photographs.

### Single nucleotide polymorphism (SNP) array

Study subjects 1 and 2 initially had SNP array analysis performed using the 250K-feature Nsp Gene-Chip according to the manufacturer's protocols (Affymetrix, Santa Clara, CA). All microarray data were MIAME-compliant and were deposited in the Gene Expression Omnibus (GEO) database (http://www.ncbi.nlm.nih.gov/geo/).

### aCGH

The deletions in study subjects 3–6 were initially identified by aCGH using various microarray platforms at Signature Genomic Laboratories. Microarray analysis using a targeted bacterial artificial chromosome (BAC)-based platform with 1887 BACs representing 622 loci was performed on DNA from study subject 4 as previously described [26]. Whole-genome BAC-based microarray analysis was originally performed on DNA from study subject 6 using a >4,600 clone custom microarray as previously described [27]. Oligonucleotide-based microarray analysis was originally performed on DNA from study subjects 3 and 5 using a custom 105K-feature whole-genome microarray (Agilent Technologies, Santa Clara, CA) as previously described [28]. In addition to the primary analysis, DNA samples from study subjects 2 to 6 were re-processed using a 244K-feature whole-genome microarray (Agilent Technologies, Santa Clara, CA) as previously described [28]. All microarray data were MIAME-compliant and were deposited in the Gene Expression Omnibus (GEO) database (http://www.ncbi.nlm.nih.gov/geo/).

### Fluorescence *in situ* hybridization (FISH)

Copy number abnormalities detected by aCGH in study subjects 3–6 were visualized by metaphase FISH using one or more BAC clones located within the abnormal regions as determined by aCGH [29]. Parental FISH testing was also performed.

### Genoglyphix Chromosome Aberration Database ™ (GCAD)

Study subjects 3–6 were identified by searching the Signature Genomic Laboratories Genoglyphix Chromosome Aberration Database (GCAD) for cases with deletions in chromosome region 20q13.33. GCAD is a database of >11,000 chromosomal abnormalities identified in more than 40,000 individuals evaluated by this laboratory from 2004 to the present.
